# Psychoactive Medication, Violence, and Variant Alleles for Cytochrome P450 Genes

**DOI:** 10.3390/jpm11050426

**Published:** 2021-05-18

**Authors:** Selma J. M. Eikelenboom-Schieveld, James C. Fogleman

**Affiliations:** 1 Independent Forensic Services LLC, 32796 Edward Dr., Conifer, CO 80433, USA; 2 Department of Biological Sciences, University of Denver, Denver, CO 80208, USA; fogleman@du.edu

**Keywords:** aggressive behavior, psychotropic drugs, CYP450 genotype, variant alleles, phenoconversion, SSRIs

## Abstract

From the start of the use of psychoactive prescription medications in the 1950s, physicians reported paradoxical adverse reactions, ranging from newly developing depressions to an increase in existing mood disorders, and extremely violent and bizarre acts of suicide and homicide. It is hypothesized that interactions between the drugs and the enzymes that are primarily responsible for their metabolism (cytochrome P450s) could cause these reactions. In this research, we evaluate statistical associations between CYP450 variant alleles, psychoactive medication, and acts of violence. Fifty-five persons who showed violent behavior or an altered emotional state were investigated for prescribed medication. Fifty-eight volunteers with no history of violence served as the controls. Genetic testing was performed on *CYP2B6*, *CYP2C8*, *CYP2C9*, *CYP2C19*, *CYP2D6* and *CYP3A4*. Statistical analysis was applied to gender, age, number of variant alleles, number and kind of medications, and potential drug–drug, drug–gene, and drug–drug–gene interactions. Four risk factors for developing an altered emotional state and/or acts of violence were identified. There is an association between prescription drugs (most notably antidepressants and other psychoactive medication), having variant alleles for CYP450 genes, and altered emotional states or acts of violence.

## 1. Introduction

Most psychoactive medication is metabolized by enzymes produced by the P450 family (CYP450 or P450). Certain allelic variants for CYP450 genes cause deviation in the rate at which prescription medication is metabolized. Most of the alternate alleles produce enzymes with reduced or no metabolic capability, although some enzymes metabolize substrates faster than normal to super-fast. Variant alleles can lead to toxic drug levels and adverse drug reactions in some patients, or to subtherapeutic levels and non-treatment responses in others [1]. By genotyping the polymorphisms at certain P450 loci, it might be possible to predict if an individual is susceptible for adverse drug reactions.

According to the FDA, psychoactive medication can cause severe side effects, varying from gastro-intestinal problems to sexual dysfunction (with both decreased and increased libido), to disorders like agitation, aggression, violence and suicidal ideation. Homicide and suicide have been reported while individuals were using antidepressants [2,3,4]. The goal of the research outlined herein is to investigate the associations between psychoactive prescription medication, P450 variant alleles, and violent behavior (assault, suicide, homicide: A/S/H) or an altered emotional state (AES), which is considered a precursor to violent behavior [5]. If an association between P450 variant alleles and medication-induced violence is established, patients at risk for side effects could possibly be identified beforehand.

This research can influence patient care, forensic science, and the justice system in several ways. Patient care is involved because CYP450 testing provides a physician with knowledge about which medication at which dose is suitable for a patient, the heart of “personalized medicine”. Forensic science could be impacted because the determination of the cause, mechanism and manner of death has far-reaching implications. In an event of alleged suicide, an association between P450 variant alleles and medication could indicate whether the medication taken was a deliberate means to suicide or that the death occurred as a result of P450 variant alleles. In the latter case, the manner of death should be ruled an accident. An association has a bearing on the justice system because, in cases of assault or homicide, it might provide an accused with a defense of involuntary intoxication or temporary insanity [6].

## 2. Materials and Methods

Between January 2018 and January 2020, Independent Forensic Services (IFS), a private forensic company (Conifer, CO, USA and Hulshorst, The Netherlands), provided de-identified case histories of individuals who have shown violent behavior or an altered emotional state, and these cases were derived from the IFS’s caseload. Several subjects were incarcerated, others had served prison time, a number of cases did not go to court and some had deceased. Case histories were included from April 2013 to April 2019. Individuals in the nonviolent group were volunteers. A data usage agreement was approved by the University of Denver’s Institutional Review Board, which approved the study protocol as well (IRB Project #910661).

Participants provided informed consent to IFS. If deceased, an appropriate relative signed a permission form. Case files of 55 persons who had shown violent behavior or an AES, were investigated for prescribed drugs. Fifty-eight normal, healthy volunteers with no history of violence were used as controls. This was used as the control group as a first step since records of potential individuals under medical care (including antidepressants) with no history of violence were not available. In the violent group, one subject was of Hispanic origin and one was of African origin. All other subjects identified as Caucasian. Because there is an essential difference between acts of violence and an altered emotional state (AES), those subgroups were also studied separately. The literature was reviewed to determine which drugs are associated with violent behavior, including illegal drugs and alcohol. In addition, the list of 350 medicines that are potentially dangerous as compiled by RxISK.org served as a guideline [7]. CYP450 testing was performed to identify variant alleles. Potential drug–drug (DDI), drug–gene (DGI), and drug–drug–gene interactions (DDGI) were noted to establish possible phenoconversion from a normal metabolizer to an intermediate, poor, or rapid phenotype [8]. Inclusion criteria for the experimental group were as follows: documented acts of violence or an altered emotional state; use of psychoactive medication around the time of an incident or in withdrawal from such medication, not exceeding a two-month period since the last use of medication; and being 18 years of age or older. The two-month time period for inclusion is because the effect of psychoactive medication on the brain can still be present weeks to months after discontinuation.

DNA sampling was done by buccal swap or a venous blood sample. CYP450 genotyping was performed either by the IFS laboratory (Hulshorst, The Netherlands), by the University of Rotterdam Erasmus Medical Center (Rotterdam, The Netherlands), or by the laboratory of OneOme (Minneapolis, MN, USA). IFS and the Erasmus Medical Center performed the genotyping using a 7500 real-time PCR system with TaqMan^®^ drug metabolism genotyping assays and software (Applied Biosystems, Waltham, MA, USA). The Erasmus Medical Center also used restriction fragment length polymorphism (RFLP), and OneOme used LGC Biosearch BHQplus^®^ assays. The following alleles were determined: CYP2B6 * 4, * 5, * 6, * 7, * 8, * 9, * 13, * 18, * 19; CYP2C8 * 3; CYP2C9 * 2, * 3;CYP2C19 * 2, * 3, * 17; CYP2D6 * 2, * 3, * 4, * 5, * 6, * 7, * 8, * 9, * 10, * 12, * 14, * 29, * 41, including copy number variants; and CYP3A4 * 1B, * 1G, * 2, * 3, * 4, * 5, * 6, * 10, * 12, * 17, * 18, * 20, * 22. The overall error rate for genotyping is 1.44% [9]. *CYP3A5* was not included due to the high (85%) sequence homology to CYP3A4 and because 80% of Caucasians (all but 2 subjects) are poor metabolizers for CYP3A5. Each allele was scored as having either normal activity, reduced activity, loss of function, or increased function. The phenotypes of the combined alleles were scored according to the criteria set by Ingelman-Sundberg [1].

Information regarding co-medication, drugs, and alcohol consumption, etc., was collected by IFS from the subjects themselves or from their medical files. Blood levels of drugs were not determined and were not sought because adverse events can occur at low to negative levels (i.e., in withdrawal). The analysis included all prescription drugs being taken by subjects, even if they were not primarily psychotropic. For this study, violence was defined according to the description used in the research of Breggin (2003), Moore et al. (2010), and Bielefeldt (2016) [2,5,10]. Included were homicide, homicidal ideation, suicide, suicidal ideation, physical assault, and violence-related symptoms, e.g., verbal abuse and/or threatening. The medical history of all participants was screened for aggressive behavior, or behavior considered a precursor for violent events. Examples of such behavior were emotional disturbances, e.g., depersonalization, disinhibition, and lack of empathy (emotional blunting). Psychotic events included abnormal feeling and thinking, nightmares, hallucinations, behavioral dyscontrol, delirium, paranoia, and mania. Jitteriness, anxiety, hyperactivity, racing thoughts, and motor restlessness were also examples of activation events as a precursor to violent events. These symptoms were indicative of an altered emotional state [5].

On a per subject basis, the DDIs, DGIs and DDGIs according to Verbeurgt et al. (2014) were established using the website Transformer [11,12,13]. For additional information and to detect differences in interpretation and in allele calls, or DDIs, DGIs, and DDGIs, the following databases were consulted: Pharmacogenomics Knowledgebase [14], FDA [15], and Pharmacogene Variation Consortium [16].

The group with violent behavior, combined with those suffering an altered emotional state, was compared with the group of healthy controls. In addition, the subgroup with violent behavior was compared to the subgroup with an altered emotional state. The parameters compared were gender, age, number of variant alleles, phenotype, number of medications, antidepressants, psychoactive medication, and number of DGI, DDI and DDGI. Regarding incidences of violence, the times spent on psychoactive medication were variable, occurred after both short and extensive time periods, and were not included in our statistical analysis. Psychiatric diagnoses were not objectifiable and were not included in the analysis. Since virtually all the data are categorical in nature, statistical testing primarily employed Chi-square statistics. When the number in any category was numerically below five, the Fisher’s exact test was performed. Statistical significance was determined with the statistical probability of 0.05 as a cutoff value.

## 3. Results

The violent group consisted of 55 individuals. Their behavior ranged from an altered emotional state (30 subjects), to assault, attempted or completed suicide and homicide (25 subjects). Two subjects in the violent group committed suicide during the research, and another died of a morphine overdose. The control group (nonviolent) consisted of 58 volunteers from the locations where IFS performs its work, both in the Netherlands and in the US. These were people without mental health issues or histories of violence. One person in this group died of natural causes.

There were 31 males and 24 females in the violent group, and 28 males and 30 females in the nonviolent group. There was no significant difference between the number of males and female participants in the different groups (*p* = 0.390). The age range in the violent group was 22–71, with a mean of 46.8 years (median = 48 years), and, in the nonviolent group, this was 18–75, with a mean of 46.9 years (median = 49 years). The age distribution between the two groups did not differ significantly.

**Figure 1:**
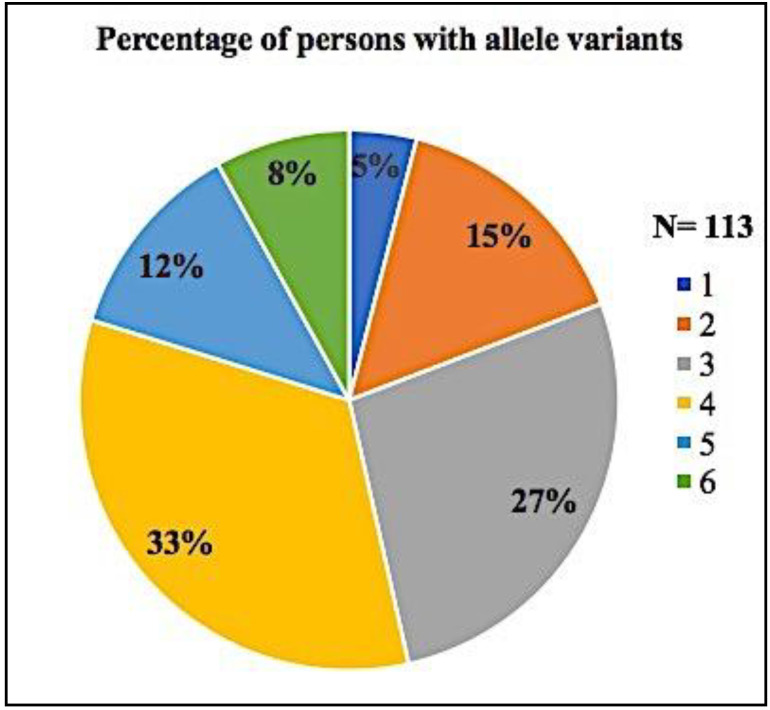
Percentage of subjects with variant alleles.

**Figure 2:**
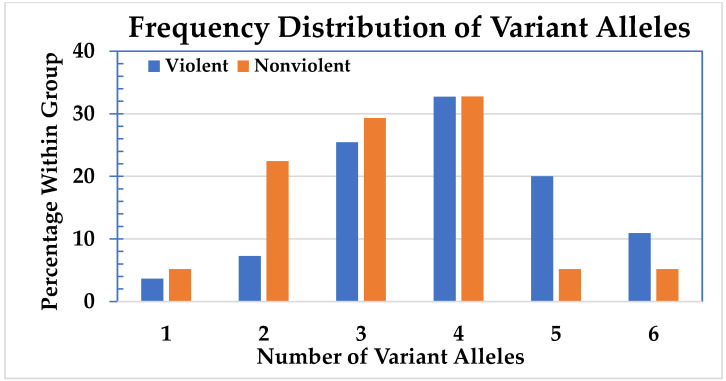
Percentage of subjects with 1–6 variant alleles.

The number of CYP450 variant alleles for all 113 subjects expressed as percentages is shown in Figure 1. The distribution of CYP450 variant alleles divided between the violent and the nonviolent group is shown in Figure 2.

Because of the low cell numbers, the subjects with one and two variant alleles were combined, as were those with five or six. The distribution of variant alleles in the groups is significantly different (*p* = 0.018). To emphasize this difference, a Chi-square test was conducted with all the cells with less than five variant alleles being summed, and the cells with five and six being summed. The result supports the hypothesis that having five or six variant alleles affects the likelihood of violent behavior (odds ratio 3.88; 95% CI: 1.40–10.76; *p* = 0.007). There are significantly more individuals with five or six variant alleles in the violent group compared to the nonviolent group.

The number of variant alleles of the CYP450s were then analyzed on a per gene basis, regardless of whether the activities of the variant alleles were reduced, loss of function, or increased (Table 1). This is appropriate because, besides high to toxic levels, fluctuations in medication levels in the blood due to P450 activity (increased or decreased) can lead to dangerous side effects as well. Only at *CYP3A4* were there significantly more variant alleles in the violent group (23) versus the nonviolent (14) (*p* = 0.045).

A similar analysis was done regarding the different CYP450s according to the metabolic capacity phenotype, as follow: extensive metabolizer (EM), intermediate metabolizer (IM), poor metabolizer (PM), rapid metabolizer or ultra-rapid metabolizer (URM). The phenotype is determined by taking the actual function of an allele into account. To distinguish the phenotypes in this research, the variant alleles that are still considered to have extensive metabolizing capacity, and therefore have the same phenotype as wildtype alleles, need to be separated from the alleles with other, altered functions. Following the allele designation of PharmVar, the distribution of phenotypes among the two groups was compared [15]. This analysis was only done for two genes, *CYP2D6* and *CYP3A4*, since these were the only two genes for which the numbers changed substantially over what is shown in Table 1. The results are presented in Table 2. The data for *CYP2D6* were analyzed using Chi-square, while the data for *CYP3A4* employed the Fisher’s exact test due to the low cell numbers involved. There was no significant difference in the phenotype between the violent and the nonviolent group regarding CYP2D6. Only with CYP3A4 were there significantly more subjects categorized as non-EM in the violent group (12) versus the nonviolent group (4) (Fisher’s exact probability = 0.031). This supports the idea that there is an association between having a CYP3A4 non-EM phenotype and violent behavior.

One additional analysis involved CYP2C19 * 17, which is an allele that results in an ultra-rapid metabolizing P450 isoform (RM or URM). There was no significant difference between the violent and nonviolent groups with respect to the (U)RM phenotype for CYP2C19 (*p* = 0.311).

**Figure 3:**
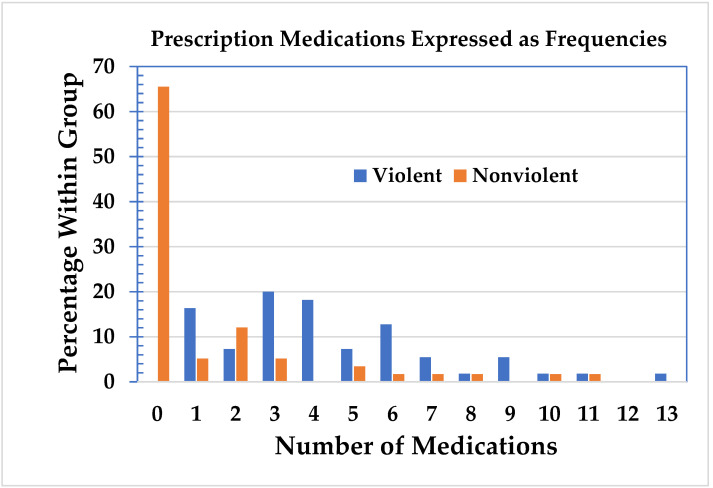
Comparison of groups with respect to the number of prescription medications.

The violent and nonviolent groups were then analyzed with respect to the number of prescription medications, including the important active metabolites, see Figure 3 [17]. In the nonviolent group, 38 subjects did not use prescription medication. In the violent group, all the subjects were on prescription medication. No subjects used hard or intravenous drugs, and three persons occasionally used cannabis, which was included in the analysis as “other” substance use. Of the 75 subjects on medication, 52 (almost 70%) were on three or more medications. The mean use of medications in the violent group was 4.4 (standard deviation = 2.77, range = 1–13, 95% confidence interval: 3.7–5.1) and 1.3 (SD = 2.56, range = 0–11, 95% CI: 0.7–2.0) in the nonviolent group. The data for both groups were separated into two or less medications and three or more medications. The Chi-square statistic was highly significant (*p* = 2.93 × 10^−10^), see Table 3. This supports the contention that there is an association between the number of medications taken and violent behavior.

The analysis was then focused specifically on the use of antidepressants within the violent and nonviolent groups. There were statistically more antidepressants used in the violent group (*p* = 9.80 × 10^−10^), see Table 4. The conclusion is that taking antidepressants affects the likelihood of violent behavior.

Further detail regarding the use of psychoactive medication was acquired by calculating the number of subjects on additional psychoactive prescription medication, e.g., antipsychotics, hypnotics (sleep medication) or mood stabilizers. In the violent group, all the subjects were on medication. In the nonviolent group, 38 subjects did not take any medication, leaving 20 subjects that did take prescription medication. In the violent group, five subjects were on antipsychotics, 21 used hypnotics and eight were prescribed a mood stabilizer. This makes a total of 34 subjects on psychoactive medication, apart from antidepressants. In the nonviolent group, there were only two subjects on hypnotics. There was significantly more psychoactive medication used in the violent group (Fisher’s exact probability = 6.09 × 10^−5^), see Table 5.

The possible associations between drug–gene, drug–drug, drug–drug–gene interactions and violent behavior were also investigated. The distribution of DGIs, DDIs and DDGIs between the violent and the nonviolent group showed no significant difference.

There were two distinct sorts of behaviors in the violent group, as follow: subjects who suffered an altered emotional state (AES, e.g., derealization, depersonalization, delusional thoughts, aggressive impulses, suicidal ideation, depression, agitation, akathisia, movement disorder, etc.) and those who exhibited real violent behavior (A/S/H, e.g., assault/suicide/homicide, including attempted suicide and homicide). There were significantly more men than women in the A/S/H subgroup (18 versus 7) compared to the AES subgroup (13 versus 17; *p* = 0.03). This supports the idea that, as far as these two subgroups are concerned, gender and violent behavior are not independent. There is no significant difference between the A/S/H and the AES subgroups regarding the number of variant alleles (*p* = 0.10), nor was there any significant difference between the number of medications between the A/S/H subgroup and the AES subgroup. As is the case with the violent and the nonviolent groups, there is a significant difference between the A/S/H and the AES subgroups regarding the use of antidepressants (*p* = 0.021, see Table 6). As can be seen, there are more A/H/S individuals using antidepressants than expected, based on random chance. Calculations were then performed with respect to the number of psychoactive prescription medications, e.g., antipsychotics, hypnotics (sleep medication) or mood stabilizers. In addition to antidepressants, three subjects in the A/S/H/ group were on antipsychotic medication, 13 were on hypnotics and three were prescribed a mood stabilizer. For the AES group those numbers were respectively two, eight and five. The Chi-square test, used to determine if the distribution of psychoactive medication between the two subgroups was comparable, showed a significant difference (*p* = 0.048), see Table 6.

The number of drug–gene interactions in the A/S/H and AES subgroups is presented in Table 7. There was no significant difference in the distribution of DGIs between the A/S/H and the AES subgroups.

**Figure 4:**
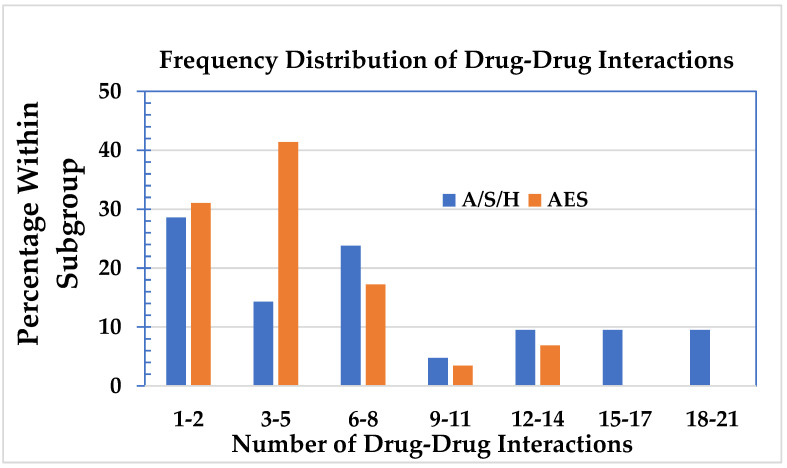
DDIs in the A/S/H and the AES subgroups.

The number of DDIs in the A/S/H and AES subgroups as frequencies is shown in Figure 4. The mean number of DDI in the A/S/H subgroup was 7.7 (SD: 6.5, range 0–21, 95% CI: 4.8–10.7) and in the AES subgroup 2.8 (SD: 1.28, range 0–17, 95% CI: 2.2–3.4). The median in the A/S/H subgroup was six, and in the AES subgroup the median was four. The average between the two, which was five, was taken to divide the A/S/H and the AES subgroups into two groups, one with five or less DDIs and one with more than five DDIs. From the data in Table 8, it appears that there are more subjects in the A/S/H subgroup with 6–21 drug–drug interactions than would be expected based on random chance (*p* = 0.037).

The mean number of DDGIs in the A/S/H subgroup was 9.32 (SD: 1.51, range 0–25) and in the AES subgroup 5.3 (SD: 0.85, range 0–12). A Chi-square test to determine if the distribution of DDGIs between the A/S/H and the AES subgroups was comparable showed no significant difference.

## 4. Discussion

Violence is a known side effect of psychoactive medication, as is recognized in the literature [2,18]. Psychoactive medication is mainly metabolized by enzymes generated by CYP450 genes [19]. Reduced or non-functional alleles will have an effect on the blood levels of drugs and can cause side effects, e.g., acts of violence.

Ji et al. (2016) concluded in their study of 1,013 subjects from the Mayo Clinic, which were genotyped for *CYP2C19*, *CYP2C9*, *CYP2D6*, *SLCO1* and *VKORC1*, that 99% of their subjects had at least one actionable variant allele [20]. In the 113 subjects of this research, 100% had at least one variant allele. Since six genes were tested, that result was to be expected. These results are consistent with those of Hocum et al. (2016) who tested over 20,000 subjects for *CYP2C19*, *CYP2C9*, *CYP2D6*, *CYP3A4* and *CYP3A5.* Only a minority had no actionable alleles for any of those CYP450 genes [21].

Although there were seven more men than women in the violent group (31 males/24 females) versus the nonviolent group (28 males/30 females), this difference was not statistically significant. However, in the analysis of the subgroups of the subjects who committed actual violence (18 males/7 females) and those who suffered an AES (13 males/17 females), the gender difference was significant. This suggests that men tend to be more aggressive than women.

Piatkov (2009), Lucire et al. (2011), Eikelenboom et al. (2016), and Ekhart et al. (2017) have studied the number of variant alleles for CYP450 genes in relation to violence and/or akathisia [4,22,23,24]. The CYP450s studied in their research were *CYP2D6*, *CYP2C9*, and *CYP2C19*. Piatkov investigated the prevalence of multiple genetic variants for *CYP2D6*, *CYP2C9* and *CYP2C19* between 546 samples of patients from the Sydney West Area Health Service (SWAHS), and 54 samples of patients with drug-induced akathisia. There was a significantly higher prevalence of multiple variant alleles among the akathisia and illicit drug users, compared to the general SWAHS population.

Lucire et al. tested 85 drug-induced akathisia patients and compared them with 150 randomly selected primary care patients. Significantly more patients in the akathisia group had at least one variant allele for the three tested CYP450s. In the studies from Piatkov and Lucire, no statistical analysis was done regarding gender, violence, medication use, any drug–drug interactions or other CYP450s involved in drug metabolism.

Ekhart et al. were unable to detect a relationship between *CYP2C19, CYP2D6* and aggression in 18 patients on SSRIs. Between 2010 and 2014, the Netherlands Pharmacovigilance Centre Lareb received 50 reports of aggressive behavior and SSRIs use. Eighteen subjects were included in the study and 20 subjects did not respond. The main argument of the authors for concluding that they could not find evidence for a significant relationship between variant alleles for *CYP2D6* and/or *CYP2C19* and aggression on SSRIs, is that, with genetic testing, no poor metabolizers (PMs) were found. However, per their own data, Ekhart et al. actually did identify two PMs. Two subjects were genotyped as CYP2D6 * 21/ * 5 and consequently phenotyped as IM. Per the PharmGKB database, the activity score for those alleles is zero, which translates into a PM phenotype [14,25]. Another subject was genotyped CYP2D6 * 21/* 41 and phenotyped EM. The activity score of * 41 is 0.5, which translates into an IM phenotype. Under “Genotyping” the authors list the genetic variants they tested for. The CYP2D6 * 21 variant is not mentioned. This illustrates the problem with rejecting an association between genetic variants and side effects based on limited genetic testing. Also, had the authors not used a platform that was able to identify the * 21, those three individuals would have been genotyped * 1/* 1 and * 1/* 41 and mis-phenotyped as EMs. Limited genetic testing likely leads to the prevalence of variant alleles being underestimated.

Many of the experiments to establish the contribution of a certain CYP450 are done in vitro, and they are generally used to guide the design of the study, which makes it hazardous to translate to human metabolism. While in vivo experiments are used to delineate the metabolism and role of various CYPs, such studies are mostly done with one substrate in a limited number of healthy volunteers over a limited period of time and often at a low dosage. This is not comparable to subjects on multiple medications over a prolonged period of time. The inter-individual expression for *CYP2C19* can vary 21- to 28-fold and, for *CYP2D6* gene expression, can vary up to 1000-fold [26]. A certain variant allele in one person can thus give a significantly different metabolic rate compared to another person with the same allele. Extrapolation from studies to individual patients or determining the metabolic rate per isolated CYP450 can create a false sense of security and provide information not consistent with the clinical practice. To get a more comprehensive picture of a subject’s metabolic capacities, it is important to genotype more than three CYP450s. If one pathway is compromised due to genetic variants, another CYP450 can take over, even if that is not the preferred pathway [19,27]. *CYP2C19* represents 3% of the total liver CYP450s and approximately 6.8% in drug metabolism, while *CYP3A4* constitutes approximately 20–50% of CYP450 content in the liver and approximately 50% to 70% of drug metabolism [26,28,29]. CYP3A4 is a high-affinity low-specificity enzyme, which can pick up the slack when other enzymes are not performing as they should [19]. Any variant allele with a reduced function would most likely be of great importance. This hypothesis was substantiated by our research results, which supports an association between having a CYP3A4 non-EM phenotype and violent behavior.

In this research, the nonviolent group appeared to be more heavily weighted for low numbers of variant alleles and the violent group appeared to be more heavily weighted for higher numbers of variant alleles. These results suggest that the subjects in the nonviolent group might be better equipped to metabolize medication than the subjects in the violent group. Twenty-three out of 113 (20%) of the subjects had five or more variant alleles for the six CYP450s tested. These subjects, irrespective of the metabolic capacity of those variants, had a greater likelihood of developing violent behavior compared to the subjects who have less than five variant alleles (odds ratio 3.9; 95% CI: 1.40–10.76).

Not only did the subjects in the violent group use significantly more medication, they also used significantly more anti-depressants (*p* = 9.80 × 10^−10^). This is in line with the current literature indicating that subjects with three or more medications, with or without depression as a possible side effect (including antidepressants), were significantly more likely to suffer depression compared to subjects not taking such drugs (15% versus 4.7%) [7,27,30]. Our results indicate that polypharmacy in general and the use of antidepressants in particular, is associated with violent behavior.

Of the six CYP450s tested, only *CYP3A4* showed more genetic variants in the violent (23) versus the nonviolent group (14, *p* = 0.045). In addition, only for CYP3A4 were significantly more subjects categorized as non-EM in the violent group (12) versus the nonviolent group (four, *p* = 0.031). Thus, only CYP3A4 was indicative for increased risk on violent behavior, both in the number of variant alleles and in the phenotyping. CYP3A4 is responsible for an estimated 50% to 70% of drug metabolism [27]. It is considered the “workhorse” and the “backup system” for the CYP450 family. For CYP3A4, there are only two phenotypes, normal and intermediate metabolizers, reported in the literature. Given that many commonly used drugs are inhibitors, substrates or inducers of CYP3A4, this enzyme is more likely to be subject to drug–drug and drug–gene interactions [19]. This makes phenoconversion a serious consideration. If a subject is an IM, or even an EM, for CYP3A4, and medication is used that inhibits this enzyme, the chances that the metabolic rate of this subject slow down to a degree that is consistent with that of a PM is not unlikely. Given the large part CYP3A4 plays in metabolizing drugs, such phenoconversion could lead to dangerous side effects, such as acts of violence.

Combining medication, and especially having three or more, can lead to drug–drug interactions, which increases the risk of dangerous side effects [30]. Drug–drug interactions (DDI) can change the expected metabolic pathways and interfere with the metabolic rate in a way comparable to drug–-gene interactions (DGI). When DDIs are superimposed on DGIs, drug–-drug–-gene interactions (DDGI) occur, increasing the effects of the DGIs and DDIs. If a subject’s metabolic rate is compromised by substrates that inhibit the expulsion of that drug from the body, and co-medication is inhibiting that process further, phenoconversion is much more likely. In a situation where there are variant alleles, which drug gets priority in being metabolized would depend on the levels of the different substrates and on the affinity of the different enzymes. In the violent group, there was only one subject who was on medication with no drug–gene interaction, leaving 54 subjects open for drug–gene interactions. In the nonviolent group, 38 subjects did not use any prescription medication, which means the control group was limited to 20 persons. The distribution of DGIs, DDIs, and DDG interactions between the violent and the nonviolent group showed no significant differences. This is most likely due to the small number of subjects in the nonviolent group, as follows: 18 with DGIs, and 11 for both DDIs and DDGIs.

The subjects committing actual violence used more medication, more antidepressants and more psychoactive medication, other than antidepressants, compared to the group of subjects suffering an AES. Since there was no difference in the number of variant alleles, this finding underscores both the importance of polypharmacy and the dangers of psychoactive medication.

With regards to the interaction between the genes and the drugs in the subgroups (A/S/H vs. AES), the distribution of the DGIs and the DDGIs were not significantly different (*p* = 0.067 and *p* = 0.159, respectively). These numbers were close to being statistically significant, indicating that the A/S/H group probably had more DD and DDG interactions, which became significant with the DDIs. There, the number of DDIs was statistically dependent on the subgroup (*p* = 0.036). This association between combining medication and side effects was not seen in the comparison between the violent and the nonviolent group. Given that the number of subjects on medication in the AES subgroup (30) is larger than the number of subjects on medication in the nonviolent medication users (18), it is understandable that a different distribution of the number of interactions between drugs and genes becomes more evident in the comparison between the A/S/H and the AES group.

The data pointed at the following two different mechanisms that could lead to medication- induced toxicity: either by way of lack of metabolic capacity through genetic variants or by the enzyme system being overwhelmed by too much medication at the same time. For the data presented herein, having more than four variant alleles was noteworthy. No distinction was made between the supposed amount of reduction or increase in the function of an allele, the only distinction was between wild type/normal function or not. As for the likelihood of developing violent behavior, increased function or decreased function did not make a difference, nor did the phenotyping for that matter, with the exception of CYP3A4.

Our results indicate that the risk factor is not the phenotype but the number of variant alleles. This could imply that, in the current system of appointing phenotypes to genotypes, the influence of variant alleles is underestimated. This applies especially to those variant alleles that lead to an IM designation and are the primary risk for phenoconversion to a PM. This is supported by the finding of Ekhart et al. that, with respect to citalopram, there were more CYP2D6 and CYP2C19 IMs than expected by random chance [24].

In an effort to apply “personalized medicine”, physicians might genotype some CYP450s. Often, these tests are limited to two or three genes. However, this will not provide enough information to know what drug–gene or drug–drug interactions to expect. Many guidelines are limited to one drug–gene interaction, and often do not address dose adjustments for IMs because they are not considered clinically important or because there are no data on the metabolic effects [31]. In this research, when the subjects in the violent and nonviolent groups are combined, 75% were on medication, and 52 of them (almost 70%) were on three or more medications. Given the extensive practice of polypharmacy, limited testing guidelines seem insufficient.

There seems to be a slippery slope in the risk for developing violent behavior. If one has a limited number of variant alleles combined with a limited amount of medication, the chances of acts of violence are also limited. This might explain why millions of people take prescription drugs and only around 1% commit acts of violence, a number the FDA nevertheless considers “frequent” [2,3,32]. When either the variant alleles or the amount of medication increase, one might develop an altered emotional state. This should be taken as a warning sign. From the medical histories, such emotional states are often considered as a sign that the medication is not working enough. A typical result is to add more or different medication, elevating a patient to a level with an increased risk of acts of violence.

The combination of five or more variant alleles, and more than two drugs, bears a serious risk of acts of violence, especially when there are antidepressants or other psychoactive medications involved. In the violent group, there were 15 subjects with the combination of three or more drugs and five or six variant alleles, and in the nonviolent group, there were only two. In the violent group, there were 12 subjects on antidepressants and who had five or six variant alleles. In the nonviolent group, there were none.

There were five subjects in the A/S/H subgroup and one in the AES subgroup who did not fit those criteria, e.g., they had either four or less variant alleles or less than three drugs. What they did have in common though was that they were put on an increasing or decreasing dose of drugs, which caused fluctuations in the drug levels in the blood. Zackrisson et al. (2009) and Ahler et al. (2010) published their research on *CYP2D6*, gene duplication, and suicide. They hypothesized that subjects with an UM phenotype might metabolize their antidepressant medication too fast and not reach the therapeutic dosage [33,34]. Another explanation could be that the rapid fluctuations in the drug levels is linked to suicidal acts. This is a known phenomenon, which is mentioned in the product labels and in the literature [35]. The FDA approved product labels for antidepressants to carry the following black box warning: “All patients being treated with antidepressants for any indication should be monitored appropriately and observed closely for clinical worsening, suicidality, and unusual changes in behavior, especially during the initial few months of a course of drug therapy, or at times of dose changes, either increases or decreases” [36].

In summary, this research identified four risk factors for acts of violence and/or an altered emotional state:

1. More than four variant alleles for the six tested CYP450s;
2. More than two drugs, especially when an antidepressant or other psychoactive medication is prescribed;
3. Having an intermediate metabolizer (IM) phenotype at CYP3A4;
4. Fluctuations in the levels of psychoactive medication in the blood.

There is an association between prescription drugs, most notably antidepressants and other psychoactive medication; having variant alleles for *CYP2B6*, *CYP2C8*, *CYP2C9*, *CYP2C19, CYP2D6 and CYP3A4*; and the occurrence of an altered emotional state or acts of violence. Based on these results, genotyping patients for these six CYP450s would provide information as to who might be susceptible to adverse drug reactions, e.g., the development of an altered emotional state or assault/suicide/homicide. This would be an improvement to personalized medicine.

To decrease the number of people dying due to acts of violence, doctors and patients need to acknowledge there is an association between psychoactive medication and acts of violence. Pathologists need to be aware that, besides toxicology screening, CYP450 testing might shed light on the actual manner of death. If a deceased has toxic levels of illicit drugs, it might be ruled an “accidental overdose”. However, if a deceased has five or more variant alleles or three or more prescribed medications, further investigation into the context of the demise is warranted before making a definite ruling of suicide. Accidental overdose could apply in those cases as well. In court cases regarding violent behavior, the genotype of a defendant is one aspect that should be taken into consideration, but it is certainly not the only one. A defendant’s medical history, the sequence of events, behavior before and after an incident, and witness statements should be put into context and explained to the judge and jury. Involuntary intoxication or temporary insanity are medical issues, whether it absolves someone of a crime is a judicial one.

In this study, the number of variant alleles were scored, indiscriminate of the amount of loss or gain of function. To be more accurate, the metabolic rate and the phenotype in cases of multiple variant alleles and polypharmacy should be measured in real life, at the time the patient is actually on the medication. Since that is not yet possible, and most variant alleles have reduced metabolic capability, the approach chosen is sufficient for the goal of this study. Also, other factors that influence the metabolic rate, e.g., co-morbidity, smoking, certain foods, were not incorporated as that information was often not available.

The drug levels in the blood at the time of the acts of violence or altered emotional states would provide important information. Unfortunately, even if blood has been drawn, it is seldom checked for more than alcohol and illegal substances. However, drug levels are not ironclad evidence, since the side effects, studied in this research, can happen at low blood levels and, when patients are in withdrawal, there would be no measurable drug levels at all [37,38,39].

The number of subjects in this study was limited (55 subjects and 58 controls). Although sufficient for statistical purposes, a larger study would provide more concrete evidence. Still, our study with 55 violent patients is a valuable addition to the research available in this field.

An often-heard argument is that an underlying psychiatric illness is responsible and that patients did not take their prescribed drugs, or not in enough quantities. Some subjects had received previous psychiatric treatment, which is not the same as suffering from a psychiatric condition. Many subjects received psychoactive medication for nonpsychiatric conditions, e.g., sleep problems, grieving, exhaustion, stress, mild anxiety, urine incontinence, or premature ejaculation. On psychoactive medication, they developed more or less severe psychiatric conditions, were diagnosed with a major depressive disorder, schizophrenia, bipolar, mania, borderline personality disorder, etc., and were completely cured after discontinuation of the prescribed medication. Such a change in mental condition does not support a diagnosis of a psychiatric illness. In addition, given the goal of this research, i.e., identifying people vulnerable to side effects, having pre-existing psychiatric conditions does not exclude them from being at risk for the effect of variant alleles or polypharmacy.

Violence, as an adverse effect, needs to be discussed between physicians and patients when one or the other is considering psychoactive medication, for whatever indication. Pharmacogenetic testing is becoming cheaper every year, and, increasingly, insurance companies are paying for it [40]. The majority of people have one or more variant alleles for the tested CYP450s, and, given that three or more drugs can put a patient at risk, pharmacogenetic testing should become standard practice. Genotyping is a powerful tool in determining metabolic rates, but there are complicating issues, e.g., substrate specificity, enzyme promiscuity, unreliability of the probe used for phenotesting, phenoconversion, heterogeneity of CYP450 enzyme activity in different organs, and altered sensitivity to inhibition. To establish causality further, prospective studies are warranted. Closely following a group of patients with known CYP450 profiles, and who are naïve in the use of psychoactive medication, could shed further light on the likelihood of developing an altered emotional state or acts of violence after starting psychoactive prescription medication. Finally, the awareness that comes with signing an informed consent, in which the side effects of acts of violence or an altered emotional state are clearly mentioned, could prevent unnecessary deaths as well.

## 5. Conclusions

- The literature review supports that psychoactive medication can cause acts of violence as an adverse drug effect;
- CYP2B6, CYP2C8, CYP2C9, CYP2C19, CYP2D6 or CYP3A4 metabolize most of the psychoactive medication;
- A group of 55 subjects who committed acts of violence was compared with 58 healthy volunteers and, within the violent group, the subjects who committed actual violence were compared to those with an altered emotional state regarding age, gender, number of variant alleles, number of medications and drug–drug interactions;
- The aim of this study was to determine whether these parameters were divided equally amongst those groups;
- The following four risk factors associated with developing an altered emotional state and/or acts of violence were identified: more than four variant alleles for *CYP2B6*, *CYP2C8*, *CYP2C9*, *CYP2C19*, *CYP2D6* or *CYP3A4*; three or more prescription medications, especially when an antidepressant or other psychoactive medication is prescribed; an intermediate phenotype for CYP3A4; and fluctuating levels of psychoactive medication in the blood;
- There is an association between prescription drugs, most notably antidepressants and other psychoactive medication; having variant alleles for *CYP2B6*, *CYP2C8*, *CYP2C9*, *CYP2C19*, *CYP2D6* and *CYP3A4*; and the occurrence of an altered emotional state or acts of violence;
- This study can influence patient care through personalized medicine, forensic science through cause, mechanism and manner of death, and the justice system by providing an accused with a possible defense of involuntary intoxication or temporary insanity;
- At present, this is the largest study in the field regarding psychoactive medication and violent behavior.

## Figures and Tables

**Table 1:** Chi-square tests of the distribution of variant alleles on a per gene basis.

Number of Variant Alleles	Violent	Nonviolent	Chi-Square Statistic (Probability)
Results for *CYP2B6*			
0	24	24	
1 or 2	31	34	0.059 (0.808)
Results for *CYP2C8*			
0	45	48	
1 or 2	10	10	0.017 (0.896)
Results for *CYP2C9*			
0	34	44	
1 or 2	21	14	2.604 (0.107)
Results for *CYP2C19*			
0	21	26	
1 or 2	34	32	0.513 (0.474)
Results for *CYP2D6*			
0	6	7	
1 or 2	49	51	0.037 (0.847)
Results for *CYP3A4*			
0	32	44	
1 or 2	23	14	4.007 (0.045) ^1^

^1^ Significant at the 5% level.

**Table 2:** Chi-square tests of the distribution of phenotypes on a per gene basis.

Phenotypes ^1^	Violent	Nonviolent	Chi-Square Statistic (Probability)
Results for CYP2D6			
EM	27	22	
Non-EM	28	36	1.432 (0.232)
Results for CYP3A4			
EM	43	54	
Non-EM	12	4	Fisher’s Exact Probability (0.031) ^2^

^1^ EM: extensive metabolizer; non-EM: non-extensive metabolizer. ^2^ Significant at the 5% level.

**Table 3:** Chi-square test of the distribution of the number of medications.

Number of Medications	Violent	Nonviolent	Chi-Square Statistic (Probability)
Less than 3	13	48	
3 or more	42	10	39.723 (2.93 × 10^−10^)

**Table 4:** Chi-square test of the use of antidepressants.

	Violent	Nonviolent	Chi-Square Statistic (Probability)
Antidepressants	35	5	
No Antidepressants	20	53	37.364 (9.80 × 10^−10^)

**Table 5:** Fisher’s exact probability test of the use of psychoactive medication.

	Violent	Nonviolent	Fisher’s Exact Probability
Psychoactive Medication	34	2	
No Psychoactive Medication	21	18	Fisher’s Exact Probability (6.09 × 10^−5^)

**Table 6:** A/H/S versus AES comparisons.

Use of Antidepressants	A/H/S	AES	Chi-Square Statistic (Probability)
Antidepressants	20	15	
No Antidepressants	5	15	5.303 (0.021)
Psychoactive Medication	19	15	
No Psychoactive Medication	6	15	3.905 (0.048)

**Table 7:** Chi-square test of the distribution of drug–gene interactions.

Number of Drug–Gene Interactions	A/S/H	AES	Chi-Square Statistic (Probability)
1–2	12	21	
3–7	13	8	3.367 (0.067)

**Table 8:** Chi-square test of the distribution of drug–drug interactions.

Number of Drug–Drug Interactions	A/S/H	AES	Chi-Square Statistic (Probability)
1–5	9	15	
6–21	12	5	4.36 (0.037)

## Data Availability

The data presented in this study are available on request from the corresponding author. The data are not publicly available due to restrictions regarding privacy.
